# Deprescribing in Community-Dwelling Older Adults: A Systematic Review and Meta-Analysis

**DOI:** 10.1001/jamanetworkopen.2025.9375

**Published:** 2025-05-08

**Authors:** Amy M. Linsky, Aneesa Motala, Marika Booth, Emily Lawson, Paul G. Shekelle

**Affiliations:** 1 Center for Health Optimization and Implementation Research, VA Boston Healthcare System, Boston, Massachusetts; 2 New England Geriatric Research Education and Clinical Center, VA Boston Healthcare System Boston, Massachusetts; 3 Department of Medicine, Boston University Chobanian & Avedisian School of Medicine, Boston, Massachusetts; 4 RAND Corporation, Santa Monica, California; 5 Southern California Evidence-based Practice Center, University of Southern California, Los Angeles; 6 Greater Los Angeles Veterans Affairs Healthcare System, Los Angeles, California

## Abstract

**Importance:**

Deprescribing has the potential to improve patient safety and quality of care by reducing polypharmacy and potentially inappropriate medications (PIMs), which in turn may reduce adverse drug events. Questions remain about the effectiveness of deprescribing interventions in outpatient settings.

**Objective:**

To determine the association of deprescribing interventions with reducing medication count and PIMs in community-dwelling older adults.

**Data Sources:**

Included studies were English-language human studies in PubMed and the Cochrane Library published from January 2019 to July 26, 2024, and results were supplemented with reference-mining and expert consultation.

**Study Selection:**

Studies were eligible if they were solely or primarily about deprescribing, focused on community-dwelling adults, were multisite, used a randomized trial design, and reported on the primary or secondary outcome.

**Data Extraction and Synthesis:**

Two authors extracted study design, intervention characteristics, population characteristics, and follow-up. Outcomes were extracted by the statistician and checked by a second author. Meta-analyses were conducted using random effects with the Hartung-Knapp-Sidik-Jonkman method. The study was reported following the Preferred Reporting Items for Systematic Reviews and Meta-analyses (PRISMA) criteria.

**Main Outcomes and Measures:**

The primary outcome was the number of PIMs or total medications, and the secondary outcome was proportion of persons prescribed at least 1 PIM.

**Results:**

Two authors independently screened 1586 titles from PubMed and Cochrane and 33 from other sources; 321 abstracts and 133 full-text studies were further reviewed, and disagreements were reconciled through discussion, resulting in 17 studies in 18 publications. A total of 8 studies of interventions targeting multiple medications were identified for primary outcome analysis; the random-effects pooled analysis found a mean difference of −0.14 (95% CI, −0.27 to −0.01) medications prescribed. A total of 6 studies of interventions targeting multiple medications were identified for secondary outcome analysis; the random effects pooled analysis found no significant reduction in the proportion of persons prescribed at least 1 PIM (odds ratio, 0.92 [95% CI, 0.74 to 1.14]).

**Conclusions and Relevance:**

This systematic review and meta-analysis found moderate-certainty evidence that deprescribing interventions were associated with reduced PIM and medication counts in community-dwelling older adults. While the individual-level association was very small, on an aggregated population level, the outcomes may be large, given the high prevalence of polypharmacy and PIMs in community-dwelling older adults.

## Key Points

**Question:**

What is the association of deprescribing interventions with reducing potentially inappropriate and total medications in community-dwelling older adults?

**Findings:**

In this systematic review and meta-analysis including 17 studies in 18 publications, there was a small reduction in medications per person (−0.14 [95% CI −0.27 to −0.01] medications). The proportion of persons with at least 1 potentially inappropriate medication was not statistically significantly lower.

**Meaning:**

This systematic review and meta-analysis found moderate certainty evidence that deprescribing interventions were associated with reducing medications in community-dwelling older adults and although the individual-level estimate was very small, the aggregated population-level effect may be large, given the high prevalence of polypharmacy in this population.

## Teaser

This systematic review and meta-analysis examines the associations of deprescribing interventions with reducing medication count and potentially inappropriate medications in community-dwelling older adults.

## Introduction

Polypharmacy (commonly defined as using ≥5 chronic medications)^1^ and potentially inappropriate medications (PIMs) are associated with adverse drug events (ADEs), increased health care utilization (eg, emergency department visits, acute care hospitalizations), and greater health care costs.^2,3,4^ These risks are especially relevant for adults aged 65 years and older, given estimates that 45% of older adults are exposed to polypharmacy^5^ and 58% to PIMs.^6^ One approach to minimize adverse outcomes is to proactively discontinue inappropriate medications. This deimplementation-based approach, known as deprescribing, is defined as a “systematic process of identifying and discontinuing drugs…[where] existing or potential harms outweigh existing or potential benefits within the context of an individual patient’s care goals.”^7^ Deprescribing has the potential to improve multiple aspects of patient safety and quality of care, including by immediately lowering drug burden, which in turn may reduce ADEs, morbidity, and mortality.^8,9^

Deprescribing interventions take many forms, including clinical pharmacist medication reviews, identifying candidate medications based on established criteria (eg, Beers, Screening Tool of Older Persons’ Potentially Inappropriate Prescriptions [STOPP]), point-of-prescribing clinical decision support [CDS], and direct-to-patient educational materials.^10,11,12,13,14^ Interventions may be isolated or longitudinal, and they may include 1 or more individuals involved in decision-making (eg, prescribers, clinical pharmacists, patients, patients’ family/caregivers).

Deprescribing spans health care settings, including outpatient clinics, acute care hospitalizations, long-term care, and community pharmacies. Adults living in the community typically have more independence managing medications, perhaps receiving assistance but not the direct administration of medications that happens in hospital or long-term care settings. As such, deprescribing interventions in this setting may function differently than in other settings. More than 90% of older adults live in the community,^15^ making this population a high priority for safe medication prescribing and use.

Given variability in deprescribing interventions, especially across care settings, questions remain about which are most effective, the best strategies to implement them, and their impact on reducing medications. This review began as part of the fourth edition of Making Healthcare Safer (MHS), evidence reviews of patient safety practices funded by the Agency for Healthcare Research and Quality (AHRQ). MHS III reviewed deprescribing and concluded that geriatrician and clinical pharmacist reviews can effectively reduce unnecessary medication use. Among the 26 eligible studies, only 5 randomized trials^16,17,18,19,20^ were conducted in community-dwelling adults; 2 studies assessed the STOPP criteria (one using an electronic health record [EHR] CDS intervention,^18^ the other using a pharmacist-led intervention^20^) and reached different conclusions, and 3 studies assessed pharmacist-led or involved interventions^16,17,19^ and found modest-to-substantial reductions in inappropriate medication use.

Since MHS III, there have been many new systematic reviews. However, these reviews have either been about specific deprescribing interventions (eg, pharmacist-delivered interventions),^11,21^ included studies across heterogenous settings (eg, hospital and long-term care settings),^22,23^ focused on health outcomes (eg, falls, mortality),^12,24^ relied on mostly observational studies as evidence,^25^ or a combination thereof. Since 2019, there have also been many published studies of deprescribing interventions in community-dwelling older adults that use a randomized clinical trial design, and thus the advisory Technical Expert Panel rated this topic a high priority for an updated review in MHS IV. Our objective with this review was to synthesize recent evidence of the association of deprescribing interventions with changes in the number of PIMs or total medications.

## Methods

This review is reported using the Preferred Reporting Items for Systematic Reviews and Meta-analyses (PRISMA) reporting guideline,^26^ and builds on work performed as a rapid response for MHS IV. Specifically, we updated the search, sharpened the inclusion criteria, and used meta-analysis to synthesize results across studies.

### Data Sources and Searches

We searched PubMed and the Cochrane Library from January 2019 (MHS III searches were through 2018) to July 26, 2024, using terms such as *deprescribing*, *polypharmacy*, and *potentially inappropriate medications*. The full search strategy is provided in eAppendix 1 in Supplement 1. We supplemented this with a search for studies via reference mining and expert consultation.

### Study Selection

Two authors (A.M.L. and P.G.S.) independently screened titles, abstracts, and full texts, with disagreements reconciled through team discussion. Studies were eligible if they were solely or primarily about deprescribing or reducing medication use, as indicated in the description of the intervention; focused on community-dwelling adults; were multisite; used a randomized trial design; and reported numeric outcomes of total medications or PIMs and/or proportions of persons with PIMs. Studies were excluded if the intervention was a more general medication review or optimization (which may result in starting new medications) or were conducted in hospital or long-term care settings. Because of the likely importance of context, we excluded studies conducted at a single site. We included interventions targeting multiple medications (ie, a large list of PIMs) and interventions targeting a single medication or class of medications (eg, Z-drugs for insomnia).

### Data Extraction and Quality Assessment

Data elements extracted in duplicate included study design, intervention characteristics, population characteristics, and follow-up. For risk of bias, we used the Cochrane Risk of Bias Tool.^27^ Data on outcomes were extracted by the statistician (M.B.) and checked by a second author (P.W.S.).

### Data Synthesis and Grading

Our primary outcome was number of prescribed medications, preferentially assessing for changes in PIMs when available. Our secondary outcome was the proportion of persons prescribed 1 or more PIMs. Both outcomes were selected given their frequent reporting and their proximal nature to the intervention (ie, more directly related, in contrast to changes in clinical outcomes). We kept separate studies targeting many medications from those targeting a single medication or class of medications because of an a priori hypothesis that deprescribing would be more effective with a narrowly targeted drug or drug class than a broad set of medications. We used the longest time of follow-up for those studies reporting multiple follow-up times. We rated certainty of the evidence using the Grading of Recommendations Assessment, Development, and Evaluation (GRADE) system.^28^

### Statistical Analysis 

A mean difference was derived for each study that reported the mean number of prescribed medications. An odds ratio (OR) was calculated when the proportion of persons was presented. We used random-effects meta-analysis using the Hartung-Knapp-Sidik-Jonkman^29,30,31,32^ method. This method has been preferred when the number of studies pooled is small and when there is evidence of heterogeneity.^32^ For the included cluster randomized clinical trials, we adjusted for clustering effects with intraclass correlation coefficient (ICC) and mean cluster size per study.^33,34^ We determined an ICC of 0.01 as the most used ICC in the included publications. We used the *I*^2^ statistic^35^ to assess the level of heterogeneity. The Egger regression asymmetry test^36^ and Begg rank correlation^37^ were used to examine publication bias. *P* values were 2-sided, and statistical significance was determined by 95% CIs that did not include the null. All analyses were conducted in R version 4.4.1 (R Project for Statistical Computing) using the metafor package. Data were analyzed from September 23 to October 14, 2024.

## Results

**Figure 1.**
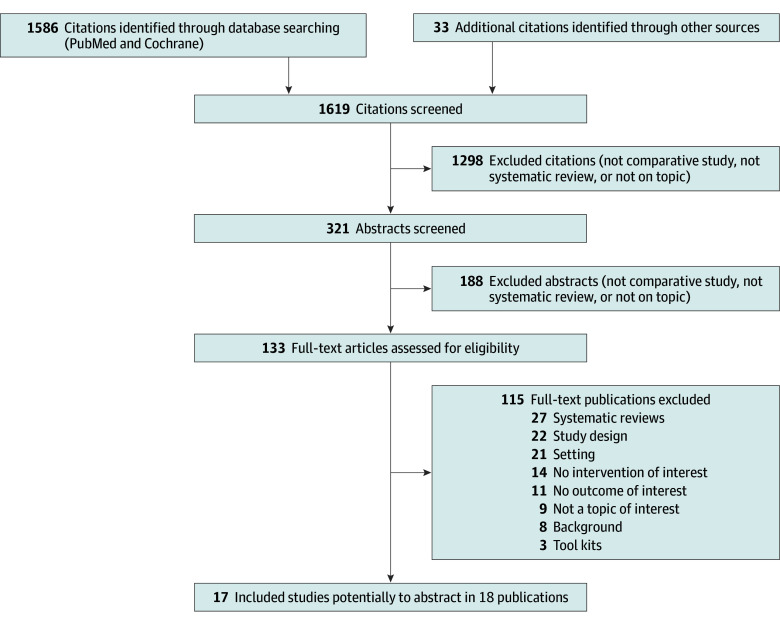
Study Selection Flowchart

After screening 1586 titles from our PubMed and Cochrane searches and adding 33 citations from other sources, we identified 133 studies for full-text screening (Figure 1). From this, there were 17 studies in 18 publications meeting all eligibility criteria (Table).^38,39,40,41,42,43,44,45,46,47,48,49,50,51,52,53,54,55^ eAppendix 2 in Supplement 1 provides a list of excluded studies, background studies, and systematic reviews.

**Table.** Details of Deprescribing Studies in Community-Dwelling Older Adults

Source	Country	Design	No. of drugs	Setting	Population	Sample size		Intervention	Comparison	Main Outcomes
						Intervention	Control			
Amorim et al,^38^ 2024	Brazil	RCT	Multiple	General practice	Older adults (age ≥60 y) receiving care in primary care facilities	7 GPs; 143 patients	7 GPs; 141 patients	Tablet-based CDS application and clinician education	Clinician education alone	Patients prescribed ≥1 PIPs at 3 mo: Intervention, 44 patients (31%) Control, 46 patients (32%) OR, 1.05 (95% CI, 0.61 to 1.81)
Bayliss et al,^39^ 2022	US	Cluster RCT	Multiple	Primary care	Age ≥65 y with dementia or mild cognitive impairment, ≥1 other chronic condition, ≥5 medications	1433 Patients in 9 clinics	1579 Patients in 9 clinics	Patient and family educational materials and clinician education materials and notifications in the EHR	UC	Long-term medications at 6 mo: Control: MD, 6.5 (95% CI, 6.4 to 6.6) medications Intervention: MD, 6.4 (95% CI, 6.3 to 6.5) medications Between-group MD, −0.10 (95% CI, −0.32 to 0.12) medications Patients prescribed ≥1 PIMs at 6 mo: Control: 330 patients (21%) Intervention: 255 patients (18%); OR, 0.82 (95% CI, 0.62 to 1.09)
Campbell et al,^40^ 2021	US	Cluster RCT	Single drug	Primary care	Age ≥65 y prescribed anticholinergics (tricyclic antidepressants and urinary antispasmodics)	254 Patients in 5 clinics	298 Patients in 5 clinics	CDS and patient educational videos	UC	Users of target anticholinergics at 12 mo: Control: 222/3004 orders (7%) Intervention: 187/3653 orders (5%) OR, 0.67 (95% CI, 0.52 to 0.86)
Harnisch et al,^41^ 2024	US	RCT	Single	Primary care	PCPs with high volume (vs peers) prescribing quetiapine for community-dwelling patients with dementia (mean age, 81 y)	33 936 Patients; 2281 PCPs	36 605 Patients; 2303 PCPs	≤3 warning letters from CMS	UC	Any receipt of quetiapine at 3 mo: MD, −1.60 (95% CI, −1.95 to −1.25)
Herrinton et al,^42^ 2023	US	RCT	Multiple	Outpatient clinics of an integrated health care system	Age ≥76 y with ≥10 nontopical prescriptions	1237 Patients	1233 Patients	Physician-pharmacist collaboration, including medication reviews and monitoring	UC, including some existing deprescribing interventions	Medications at 6 mo: Control (n = 1233): MD, −0.4 (95% CI, −0.6 to −0.3) medications Intervention (n = 1062): MD, −0.4 (95% CI, −0.6 to −0.2) medications Between-group MD, 0.00 (95% CI, −0.28 to 0.28) medications
Jamieson et al,^43^ 2023; Nishtala,^50^ 2023	New Zealand	RCT	Multiple	Community- dwelling adults getting care from GPs	Age ≥65 y taking ≥1 anticholinergic or sedative drug	184 Patients	179 Patients	Pharmacist-conducted medication review, patient education, and letter to GP with suggestions for deprescribing	UC	Patients with a reduction in DBI ≥0.5 at 6 mo: Control (n = 179): 21 patients (13%) Intervention (n = 171): 21 patients (12%) OR, 1.05 (95% CI, 0.56 to 1.98)
Kouladjian O’Donnell et al,^44^ 2021	Australia	Cluster RCT	Multiple	Community-dwelling adults nationwide	Age ≥65 y and eligible for a home medication review	88 Patients; 29 pharmacists	113 Patients; 24 pharmacists	Pharmacist-led use of computerized CDS system, with results sent to the GP and to patient (or caregiver)	Pharmacist-conducted home medication review without CDS system	Mean change in DBI from baseline to 3 mo: Control (n = 96): 0 (95% CI, −0.22 to 0.22) Intervention (n = 63): −0.02 (95% CI, −0.44 to 0.40) Patients with reduced DBI at 3 mo: Control: 11 patients (11%) Intervention: 11 patients (17%) OR, 0.61 (95% CI, 0.24 to 1.53)
Kuntz et al,^45^ 2019	US	RCT	Single	Community-living adults of an integrated care delivery system	Age ≥64 y and prescribed a Z-drug (eszopiclone, zolpidem, or zaleplon)	Education and prescriber letter: 50 patients; intervention + pharmacist counseling session: 49 patients	50 Patients	Patient education and letter from prescriber, with or without pharmacist telephone counseling session	UC	Z-drugs dispensed at 6 mo: Education + letter: MD (SD), 1.9 (1.6) dispensings Education + letter + counseling: MD (SD), 1 (1.6) dispensings Between-group MD, −0.90 (95% CI, −1.53 to −0.27) dispensings Discontinuation rate at 6 mo: Education + letter: 13 patients (26%) Education + letter + counseling: 27 patients (55%) OR, 0.29 (95% CI, 0.12 to 0.67)
Mahlknecht et al,^46^ 2021	Italy	Cluster RCT	Multiple	General practice	Age ≥75 y and receiving ≥8 prescribed active agents	281 Patients; 22 GPs	257 Patients; 21 GPs	Expert-led drug review with deprescribing recommendations sent to the GP	UC	Mean change in No. of drugs since baseline at 24 mo: Intervention (n = 235): 0 (95% CI, −2 to 0) drugs Control (n = 250): MD, 0 (95% CI, −1 to 0) drugs Between-group MD, 0 (95% CI, −0.22 to 0.22) drugs
Mak et al,^47^ 2024	US	RCT	Single	VA patients in 2 states	Age ≥65 y who received ≥1 benzodiazepine-receptor agonist in the prior 18 mo	Brochure: 946 patients; brochure + telephone: 120 patients	943 Patients	Educational brochure with or without telephone reinforcement	Control brochure	BZRA within 90 d of follow-up at 12 mo: OR, 1.16 (95% CI, 0.78 to 1.73)
McCarthy et al,^48^ 2022	Ireland	Cluster RCT	Multiple	General practice	Age ≥65 y and ≥15 regular medications	217 Patients; 26 GPs	205 Patients; 25 GPs	Web-based CDS system for medication review	UC	PIPs at 6 mo: Control (n = 196): MD (SD), 2.35 (1.43) PIPs Intervention (n = 208): MD (SD), 2.16 (1.44) PIPs Between-group MD, −0.19 (95% CI, −0.48 to 0.10) PIPs Patients with ≥1 PIPs at 6 mo: Control: 179 patients (92%) Intervention: 181 patients (87%) OR. 0.62 (95% CI, 0.32 to 1.23)
Mortsiefer et al,^49^ 2023	Germany	Cluster RCT	Multiple	General practice	Age ≥70 y, evidence of frailty, and ≥5 drugs/d	272 Patients; 59 GPs	249 Patients; 54 GPs	GP education and 3 in-home family conferences, including a medication review	UC	PIMs at 12 mo: Control (n = 171): MD (SD), 1.64 (1.15) PIMs Intervention (n = 176): MD (SD), 1.45 (1.21) PIMs Between-group MD, −0.19 (95% CI, −0.44 to 0.06) PIMs
Phelan et al,^51^ 2024	US	Cluster RCT	Single	Primary care	Age ≥60 y and using opioids or sedative-hypnotics; age ≥65 y using muscle relaxants, tricyclic antidepressants, or first-generation antihistamines	1106 Patients	1261 Patients	Patient education and CDS	UC	Discontinuation rate 90 d after follow-up at 15 mo: Control: 227 patients (18%) Intervention: 232 patients (21%) OR, 0.83 (95% CI, 0.61 to 1.13)
Rieckert et al,^52^ 2020	Europe	Cluster RCT	Multiple	General practice	Age ≥75 y and ≥8 drugs	1953 Patients; 181 general practices	1951 Patients; 178 general practices	Computerized CDS	UC	Prescribed drugs at 24 mo: Intervention (n = 1953): MD (SD), 10.52 (2.94) drugs Control (n = 1953): MD (SD), 10.12 (3.01) drugs Between-group MD, −0.40 (95% CI, −0.60 to −0.20) drugs
Rudolf et al,^53^ 2021	Germany	Cluster RCT	Multiple	Primary care	Age ≥70 y and ≥6 drugs for regular long-term use	Intervention 1: 285 patients; 34 practices; intervention 2: 291 patients; 35 practices	562 Patients; 68 practices	Intervention 1: physician education alone; intervention 2: physician and team training	Physicians invited to a workshop on general aspects of geriatric pharmacotherapy	Patients with ≥1 PIMs at 12 mo: Control: 124/478 patients (26%) Combined interventions: 136/491 patients (28%) OR, 1.09 (95% CI, 0.82 to 1.47)
Wallis et al,^54^ 2022	New Zealand	Cluster-RCT	Single	General practice	Patients at increased risk for harms from NSAIDs, based on age, kidney disease, heart failure, or on other antiplatelet/anticoagulants	11 658 Patients; 20 practices	10 209 Patients; 19 practices	Physician education, pharmacist outreach, letter from GPs to patients about discussing medications	UC	Users of high-risk prescriptions (NSAIDs and/or anticoagulants) at 12 mo: Control: 419/9199 patients (5%) Intervention: 538/9453 patients (6%) OR, 1.25 (95% CI, 1.00 to 1.57)
Zechmann et al,^55^ 2020	Switzerland	Cluster RCT	Multiple	Primary care	Age ≥60 y and ≥5 chronic drugs	128 Patients; 22 physicians	206 Patients; 24 physicians	Medication review between physician and patient using shared decision-making	UC	Drugs per person at 12 mo: Control (n = 190): MD, 7.68 (95% CI, 7.32 to 8.00) drugs Intervention (n = 115): MD, 7.64 (95% CI, 7.17 to 8.12) drugs Between-group MD, −0.04 (95% CI, −0.65 to 0.57) drugs

Abbreviations: CDS, clinical decision support; CMS, Centers for Medicare & Medicaid Services; GP, general practitioner; ICU, intensive care unit; MD, mean difference; NSAID, nonsteroidal anti-inflammatory drug; OR, odds ratio; PCP, primary care practitioner; PIM, potentially inappropriate medication; PIP, potentially inappropriate prescription; RCT, randomized clinical trial; UC, usual care.

### Description of the Evidence

Of 17 studies,^38,39,40,41,42,43,44,45,46,47,48,49,51,52,53,54,55^ 11 were cluster-randomized trials.^39,40,44,46,48,49,51,52,53,54,55^ All but 1 study^54^ enrolled participants aged at least 60 years, and all studies specified participants as attending primary care, general practice, or as community-dwelling adults. Sample sizes ranged from an intervention in 7 general practitioner (GP) practices with 143 participants^38^ to 2281 primary care practitioners and 33 936 participants,^41^ although most studies had between 10 and 25 practices and 100 and 500 participants. The mean (SD) number of baseline medications per participant was 9.74 (2.54). Most interventions were aimed at health care practitioners and many of these included a component of patient education, but 2 studies had interventions aimed exclusively at patients.^45,47^ Thirteen studies^39,40,41,42,43,45,46,48,49,51,52,54,55^compared the intervention to usual care, while the remaining studies compared a multicomponent intervention to clinician education alone,^38^ pharmacists’ medication review with computerized CDS vs medication review alone,^44^ a patient education brochure vs a control brochure,^47^ and physician education vs a control educational class.^53^

### Risk of Bias Assessment

The assessments of the Cochrane Risk of Bias criteria are in eAppendix 3 and eTable 1 in Supplement 1. Most clinician-focused interventions are challenging to blind, and our assessments of the risk of bias for that domain reflect this. If the number of medications prescribed was ascertained from pharmacy data in the EHR or administrative data, we judged that to be low risk of bias; if data came from patient or clinician report of medications, then the study was judged to have high risk of bias. All studies were selected because they were randomized trials of deprescribing interventions reporting the outcome of medication use, and thus the domains of randomization and selective outcome reporting were uniformly judged as low risk of bias. All but 6 studies^38,41,43,47,51,55^ were judged to be at high risk of bias in at least 1 domain. Two studies^41,47^ were low risk of bias in all domains.

### Studies Reporting the Number of Prescribed Medications

**Figure 2.**
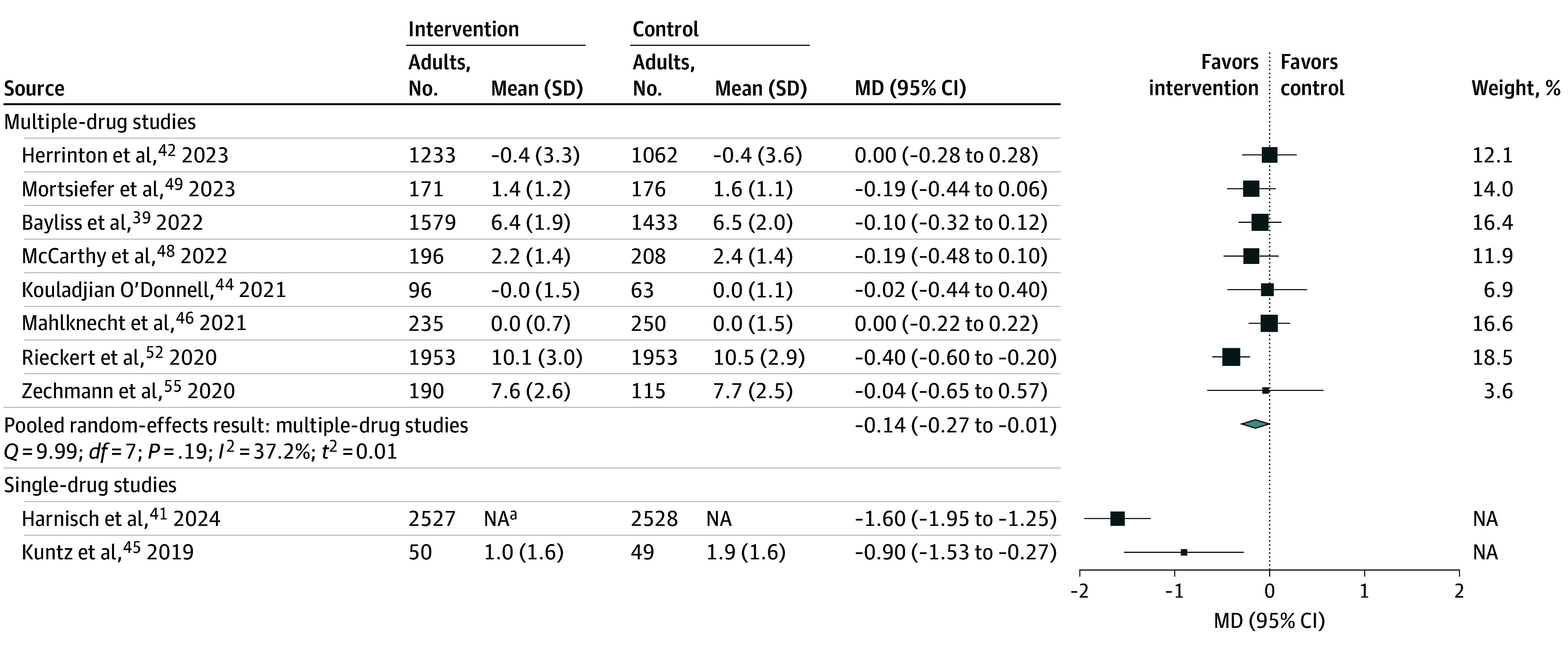
Findings of Interventions for the Number of Prescribed Medications Sizes of squares indicate weight of studies; MD, mean difference; NA, not applicable. ^a^Reported mean difference, not report mean (SD) by group.

We identified 10 studies^39,41,42,44,45,46,48,49,52,55^ of interventions that reported the number of medications (Figure 2), 3 of which^44,48,49^ specifically reported PIMs. Eight of these studies^39,42,44,46,48,49,52,55^ had interventions targeting deprescribing of multiple medications. Of the these 8 studies, 2 studies^39,42^ were conducted in the US, 1 study each was conducted in Germany,^49^ Ireland,^48^ Italy,^46^ Australia,^44^ and Switzerland,^55^ and 1 study^52^ was a multinational European study. The random-effects pooled estimate for these 8 studies was a mean difference in the number of PIMs or total medications prescribed of −0.14 (95% CI, −0.27 to −0.01) medications (*I*^2^ = 37%). There was no statistical evidence of publication bias (Egger test: *P* = .51; Begg test: *P* = .90). The study reporting the most favorable estimate (standardized mean difference of −0.4) tested an electronic CDS tool in the general practices of 4 European countries.^52^ In the 3 studies with the least favorable estimate (point estimate ≤0.02), one study assessed a pharmacist use of an electronic CDS in Australia,^44^ a second study used a physician-pharmacist collaboration that included medication reviews and monitoring,^42^ and the third study tested an expert review of medications with deprescribing recommendations that was communicated to the treating GP in Italy.^46^ Four^42,46,52,55^ of the 8 studies reported medications most often identified as deprescribing targets; these included proton pump inhibitors, H2 receptor antagonists, anxiolytics/hypnotics, antidepressants/antipsychotics, antihypertensives, aspirin, and simvastatin.

Two studies reported the outcome of reduction in prescribed medications for interventions targeting single drugs or drug classes. One US-based study^41^ targeted quetiapine in people with dementia and found that receipt of up to 3 overprescribing warning letters was associated with 1.5 (95% CI, −1.8 to −1.1) fewer days of quetiapine use. A second US-based study targeted nonbenzodiazepine sedatives (ie, Z-drugs) and found that an intervention of pharmacist telephone consultation and patient education was associated with 55% of patients discontinuing the use of Z-drugs, compared with 26% of patients receiving usual care.^45^

### Studies Reporting the Proportion of Patients Prescribed 1 or More PIMs

**Figure 3.**
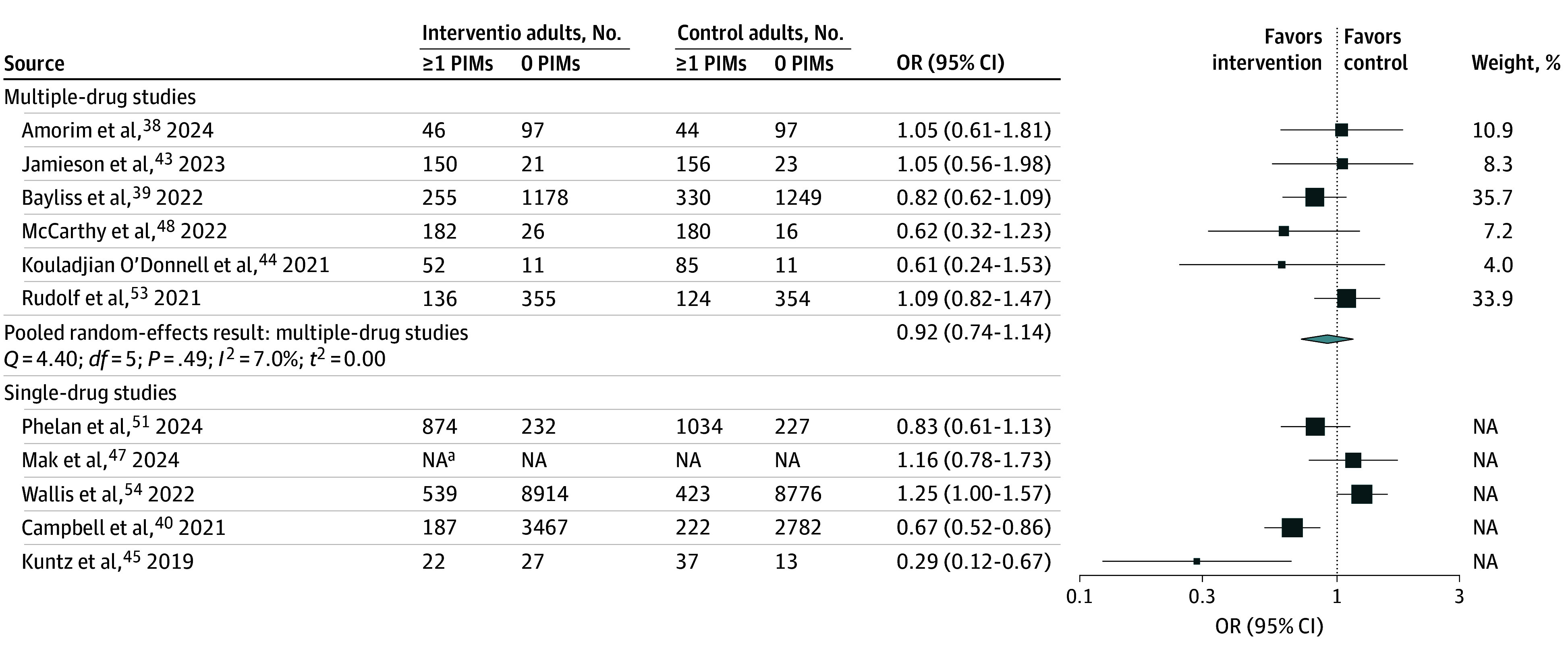
Forest Plot of Deprescribing Interventions on the Proportion of Patients Prescribed 1 or More Potentially Inappropriate Medications (PIMs) Sizes of squares indicate weight of studies; NA, not applicable; OR, odds ratio. ^a^Reported OR, not counts by group.

We identified 11 studies^38,39,40,43,44,45,47,48,51,53,54^ of interventions that reported the proportion of patients prescribed 1 or more PIMs (Figure 3). Six of these studies targeted deprescribing in general^39,44,48^ or specific lists of drugs, such as PIMs.^38,43,53^ Each of the 6 studies was conducted in a different country: the US,^39^ Ireland,^48^ Australia,^44^ Germany,^53^ Brazil,^38^ and New Zealand.^43^ The random effects pooled analysis found no significant reduction in the proportion of persons prescribed at least 1 PIM (OR, 0.92 [95% CI, 0.74 to 1.14]; *I*^2^ = 7%). There was no statistical evidence of publication bias (Egger test: *P* = .64; Begg test: *P* = .72). The study with the most favorable point estimate of effect (OR, 0.62 [95% CI, 0.24-1.23]) tested the use of a custom-designed web-based education and CDS among GPs in Ireland.^48^ The study reporting the least favorable estimate tested a clinician education intervention in German primary care practices.^53^

We found 5 studies^40,45,47,51,54^ that reported the proportion of patients prescribed 1 or more PIMs with an intervention targeting a single medication or medication class. Four studies^40,45,47,51^ were from the US; the remaining study was conducted in New Zealand.^54^ The medications or medication classes targeted were benzodiazepines, Z-drugs, or other central nervous system–acting medications^45,47,51^; nonsteroidal anti-inflammatory drugs and other antiplatelet agents^54^; and medications with anticholinergic effects.^40^ Intervention components were heterogeneous and included EHR-based decision support, pharmacist consultation, clinician education, and patient education. Of these 5 studies, 3 studies^40,45,51^ reported outcomes favoring the intervention, while 2 studies^47,54^ reported effects favoring the control group; in 1 study, the difference was statistically significant. The heterogeneity of the results precluded formal meta-analyses, and do not support the a priori hypothesis of greater effectiveness of deprescribing interventions in narrowly targeted medications.

### Certainty of Evidence

We judged the certainty of evidence that deprescribing interventions can reduce number of PIMs or total medications in community-dwelling older adults as moderate, downgraded from high due to inconsistency in results, possibly due to heterogeneity in the interventions or the contexts in which they were tested (eTable 2 in Supplement 1). Any association with reducing the proportion of patients prescribed at least 1 PIM is very low certainty because the pooled analysis, although in the right direction, did not find a statistically significant difference.

## Discussion

In this systematic review and meta-analysis, we found moderate certainty evidence that deprescribing interventions were associated with reductions in the mean number of medications prescribed to community-dwelling older adults with polypharmacy. However, the estimate was small: on average, approximately 7 patients would need to be exposed to the intervention to get a reduction in 1 prescribed medication. Given that the mean number of baseline medications in the included studies was 9.74, the total number of medications prescribed across 7 typical patients would decrease from 68 to 67. At the individual level, this benefit is very small. Yet, if the usual primary care clinician has a panel of 2000 patients and at least half of those patients meet the definition of polypharmacy, then a deprescribing intervention applied at the practice level would be expected to reduce the number of prescribed drugs by at least 140 medications. Thus, the deprescribing interventions that have been tested in community-dwelling older adults are conceptually similar in benefit to cancer screening interventions, where the individual benefit is very small, but because such a large population is eligible for the intervention, the population benefit is moderate or even large.

The observation that some systematic reviews^12,21,24^ found no associations of deprescribing interventions with health outcomes can be interpreted in the same manner as a noninferiority trial. That is, clinical outcomes, such as mortality, are unchanged in the context of fewer medications, suggesting minimal or no harm results from deprescribing. Rather, it is possible that reduction in number of medications has small direct benefits to individuals, such as decreased pill burden and out-of-pocket costs. Aggregated at the population level, reduced pharmacy-related spending can allow reallocation of resources to high-value care. An additional potential benefit of deprescribing, although not established, is population-level reductions in adverse drug events, especially if targeted medications are those with higher risk of causing harm.

We hypothesized that interventions targeting a single medication or medication class would be more effective, perhaps by allowing both clinicians and patients to pay greater attention to potential risks and benefits of just 1 medication. Our review identified too few studies of single-medication deprescribing interventions to conduct a meta-analysis. While the 2 trials reporting on the outcome of number of medications demonstrated a greater magnitude of effect, aligning with our a priori hypothesis that deprescribing interventions are more effective when targeting a narrow set of medications, the 5 studies reporting on proportion of patients with PIMs had inconsistent findings. Additional trials are needed to determine the comparative effectiveness of interventions focused on single medications compared with interventions targeting multiple medications.

### Limitations

Our study has some limitations, which can be classified into 2 categories, the first based on the source material and the second resulting from our methods. Regarding source material limitations, our reliance on randomized trials substantially mitigates the bias introduced by using observational study data; however, most included studies still had at least 1 domain judged to be at high risk of bias, mostly due to challenges blinding the participants of a deprescribing intervention. A second limitation of the source material is the heterogeneity of the interventions, such that the identical intervention was never the subject of more than 1 study. Thus, there may be components of the intervention or of the context that are more or less important in determining effectiveness but which cannot currently be assessed due to lack of sufficient data. Regarding our review methods, the principal threat is that we did not identify all eligible studies, either because we did not search broadly enough or because some deprescribing interventions that have been implemented and assessed for effectiveness never had their results published. The effect of such missing data on our findings is speculative.

## Conclusions

This systematic review and meta-analysis found moderate certainty evidence that deprescribing interventions were associated with reduced polypharmacy and PIM use in community-dwelling older adults. While the outcomes at the individual level were very small, on an aggregated population level, these differences may be large, given the high prevalence of polypharmacy and PIM use in community-dwelling older adults.

## Notes

Supplement 1. **eAppendix 1.** Search Strategy **eAppendix 2.** Excluded Studies, Background Studies, and Systematic Reviews **eAppendix 3.** Cochrane Risk of Bias for RCTs **eTable 1.** Cochrane Risk of Bias for RCTs **eTable 2.** GRADE Certainty of Evidence Table
eReferences.

Supplement 2.
Data Sharing Statement
