# Supplementary material for: Deprescribing in Community-Dwelling Older Adults: A Systematic Review and Meta-Analysis
Source: JAMA Netw Open. 2025 May 8;8(5):e259375. doi: 10.1001/jamanetworkopen.2025.9375 (PMC12062908; doi:10.1001/jamanetworkopen.2025.9375)
Supplement: Supplement 1. — eAppendix 1. Search Strategy eAppendix 2. Excluded Studies, Background Studies, and Systematic Reviews eAppendix 3. Cochrane Risk of Bias for RCTs eTable 1. Cochrane Risk of Bias for RCTs eTable 2. GRADE Certainty of Evidence Table eReferences. [file jamanetwopen-e259375-s001.pdf]

Supplemental Online Content

Linsky AM, Motala A, Booth M, Lawson E, Shekelle PG. Deprescribing in community-dwelling older adults: a systematic review and meta-analysis. *JAMA Netw Open*. 2025;8(5):e259375. doi:10.1001/jamanetworkopen.2025.9375

eAppendix 1. Search Strategy
eAppendix 2. Excluded Studies, Background Studies, and Systematic Reviews
eAppendix 3. Cochrane Risk of Bias for RCTs
eTable 1. Cochrane Risk of Bias for RCTs
eTable 2. GRADE Certainty of Evidence Table
eReferences.

This supplemental material has been provided by the authors to give readers additional information about their work.

© 2025 Linsky AM et al. *JAMA Network Open*.

Supplemental File 1. Search Strategy

Databases:

- PubMed (NIH/NLM)
- Cochrane Library

Limits:

- 2019 – 2024
- In English

Deliverables:

- Word doc of all search strategies
- De-duplicated EndNote library of all search results

Results:

- Total # for review post-deduplication of EN Library: **1,584**

PubMed

Set #	Search	# of results
**1**	elder[tiab] OR elders[tiab] OR elderly[tiab] OR geriatric*[tiab] OR gerontolog*[tiab] OR “old age”[tiab] OR “oldest old”[tiab] OR “senior citizen*”[tiab] OR “very old”[tiab] OR septuagenarian*[tiab] OR octogenarian*[tiab] OR octagenarian*[tiab] OR nonagenarian*[tiab] OR centarian*[tiab] OR centenarian*[tiab] OR supercentenarian*[tiab] OR “older people”[tiab] OR “older person”[tiab] OR “older subject*”[tiab] OR “older patient*”[tiab] OR “older age*”[tiab] OR “older adult*”[tiab] OR “older man”[tiab] OR “older men”[tiab] OR “older male*”[tiab] OR “older woman”[tiab] OR “older women”[tiab] OR “older female*”[tiab] OR “older veterans”[tiab] OR “older population”[tiab] OR "nursing home*"[tiab] OR “Aged”[MAJR] OR "Aged, 80 and over"[MAJR] OR “Health Services for the Aged”[MAJR] OR “Geriatric Assessment”[MAJR] OR “Geriatrics”[MAJR] OR “Geriatric Psychiatry”[MAJR] OR “Homes for the Aged”[MAJR] OR “Nursing Homes”[MAJR]	728,186
**2**	(“deprescrib*”[tiab] OR “de prescribing”[tiab] OR “de prescription*”[tiab] OR “inappropriate prescri*”[tiab] OR “inappropriate medication*”[tiab] OR "potentially inappropriate*"[tiab] OR PIMs[tiab] OR PIM[tiab] OR "appropriateness medications"[tiab:~2] OR "appropriate prescribing"[tiab] OR ((“medication*”[ti] OR “prescribing”[ti] OR prescription*[ti]) AND (inappropriate*[ti] OR “over prescrib*”[ti] OR overus*[ti])) OR “polypharmacy”[tiab] OR ((medication*[ti] OR drugs[ti] OR "drug therap*"[ti] OR prescription*[ti] OR “prescribing”[ti]) AND (inappropriate*[ti] OR appropriate*[ti]) AND (reduc*[ti] OR discontinu*[ti] OR withdraw*[ti] OR cease[ti] OR ceasing[ti] OR cessation[ti] OR “dose reduction”[ti] OR taper*[ti])) OR ((review*[ti] OR assess*[ti] OR evaluat*[ti] OR screen*[tiab] OR STOPP[tiab] OR START[tiab] OR "STOPP START"[tiab:~1] OR "Beers Criteria"[tiab]) AND (appropriate*[tiab] OR inappropriate*[tiab]) AND ("drug utilization"[ti] OR medication[ti] OR prescrib*[ti] OR prescription*[tiab] OR "drug	30,138

© 2025 Linsky AM et al. *JAMA Network Open*.

	therap*"[ti])) OR "geriatric pharmacotherap*"[tiab] OR "geriatric pharmacol*"[tiab] OR "Deprescriptions"[MAJR] OR "Inappropriate Prescribing"[MAJR] OR "Drug Utilization Review"[MAJR:NoExp] OR Polypharmacy[MAJR] OR "Potentially Inappropriate Medication List"[MAJR])	
**3**	(#1 AND #2) AND ((2023/7/1:2024/12/31[pdat]) AND (english[Filter]))	1,072
**4**	clinicalstudy[Filter] OR clinicaltrial[Filter] OR comparativestudy[Filter] OR controlledclinicaltrial[Filter] OR evaluationstudy[Filter] OR governmentpublication[Filter] OR observationalstudy[Filter] OR randomizedcontrolledtrial[Filter] OR researchsupportamericanrecoveryandreinvestmentact[Filter] OR researchsupportnihextramural[Filter] OR researchsupportnihintramural[Filter] OR researchsupportusgovtnonphs[Filter] OR researchsupportusgovtphs[Filter] OR researchsupportusgovernment[Filter] OR validationstudy[Filter] OR "randomized control*"[ti] OR "randomised control*"[ti]	6,225,334
**5**	#3 AND #4	**127**
**6**	systematicreview[Filter] OR meta-analysis[Filter] OR review[Filter] OR guideline[Filter] OR practiceguideline[Filter] OR "systematic review"[ti] OR meta-analysis[ti] OR "scoping review"[ti] OR "state of the art"[ti] OR "realist review"[ti] OR "rapid review"[ti] OR "narrative review"[ti] OR "umbrella review"[ti] OR "rapid evidence"[ti] OR "literature review"[ti] OR "evidence synthesis"[ti] OR "evidence summary"[ti] OR guideline*[ti]	3,725,993
**7**	#5 AND #6	**6**

References:

Deprescribing Literature Search Strategy - US Deprescribing Research Network

Development and validation of search filters to identify articles on deprescribing in Medline and Embase | BMC Medical Research Methodology | Full Text (biomedcentral.com)

A systematic review of the emerging definition of 'deprescribing' with network analysis: implications for future research and clinical practice - PubMed (nih.gov)

Cochrane Library: Cochrane Database of Systematic Reviews (CDSR), the Cochrane Central Register of Controlled Trials (CENTRAL), Cochrane Clinical Answers

Set #	Search	# of results
**1**	deprescrib*:ti,ab,kw OR "de prescribing":ti,ab,kw OR deprescription*:ti,ab,kw OR ("inappropriate" NEAR prescri*):ti,ab,kw OR "inappropriate medication":ti,ab,kw OR ("potentially inappropriate" NEAR medication*):ti,ab,kw OR PIMs:ti,ab,kw OR PIM:ti,ab,kw OR ("appropriateness" NEAR medication*):ti,ab,kw OR "drug utilization review":ti,ab,kw OR "appropriate prescribing":ti,ab,kw OR polypharmacy:ti,ab,kw OR ("geriatric" NEAR pharmacotherap*):ti,ab,kw OR ("geriatric" NEAR pharmacol*):ti,ab,kw OR ((medication*:ti OR prescribing:ti OR prescription*:ti) AND (inappropriate*:ti OR ("over" NEAR prescrib*):ti OR overus*:ti)) OR ((medication*:ti OR drugs:ti OR "drug therapy":ti OR prescription*:ti OR prescribing:ti) AND (inappropriate*:ti OR appropriate*:ti) AND (reduc*:ti OR discontinu*:ti OR withdraw*:ti OR cease:ti OR ceasing:ti OR cessation:ti OR "dose reduction":ti OR taper*:ti)) OR ((review*:ti OR assess*:ti OR	3,282

© 2025 Linsky AM et al. *JAMA Network Open*.

	evaluat*:ti OR screen*:ti OR STOPP:ti,ab,kw OR START:ti,ab,kw OR "STOPP START":ti,ab,kw OR "Beers Criteria":ti,ab,kw) AND (appropriate*:ti,ab,kw OR inappropriate*:ti,ab) AND ("drug utilization":ti OR medication:ti OR prescrib*:ti OR prescription*:ti OR "drug therapy":ti))	
**2**	#1<br>Limits: July 2023- Dec 2024 (07/01/2023 – 31/12/2023); Cochrane Reviews	**3**

© 2025 Linsky AM et al. *JAMA Network Open*.

Supplemental File 2. Excluded Studies, Background Studies, and Systematic Reviews

Excluded Studies

1. Impact of deprescribing for people with limited life expectancy. Drug Ther Bull. 2023 May;61(5):69. doi: 10.1136/dtb.2023.000012. PMID: 36882298. *Design*

2. Alharthi M, Wright D, Scott S, et al. Barriers and enablers to deprescribing for older people in care homes: The theory-based perspectives of pharmacist independent prescribers. Res Social Adm Pharm. 2023 May;19(5):746-52. doi: 10.1016/j.sapharm.2023.01.013. PMID: 36732210. *Design*

3. Aubert CE, Blum MR, Gastens V, et al. Prescribing, deprescribing and potential adverse effects of proton pump inhibitors in older patients with multimorbidity: an observational study. CMAJ Open. 2023 Jan-Feb;11(1):E170-e8. doi: 10.9778/cmajo.20210240. PMID: 36854455. *Design*

4. Balsom C, Pittman N, King R, et al. Impact of a pharmacist-administered deprescribing intervention on nursing home residents: a randomized controlled trial. Int J Clin Pharm. 2020 Aug;42(4):1153-67. doi: 10.1007/s11096-020-01073-6. PMID: 32494991. *Setting*

5. Baumgartner AD, Clark CM, LaValley SA, et al. Interventions to deprescribe potentially inappropriate medications in the elderly: Lost in translation? J Clin Pharm Ther. 2020 Jun;45(3):453-61. doi: 10.1111/jcpt.13103. PMID: 31873955. *Outcome*

6. Bighelli I, Rodolico A, Siafis S, et al. Antipsychotic polypharmacy reduction versus polypharmacy continuation for people with schizophrenia. Cochrane Database of Systematic Reviews. 2022(8). doi: 10.1002/14651858.CD014383.pub2. PMID: CD014383. *Intervention*

7. Blum MR, Sallevelt B, Spinewine A, et al. Optimizing Therapy to Prevent Avoidable Hospital Admissions in Multimorbid Older Adults (OPERAM): cluster randomised controlled trial. Bmj. 2021 Jul 13;374:n1585. doi: 10.1136/bmj.n1585. PMID: 34257088. *Setting*

8. Boersma MN, Huibers CJA, Drenth-van Maanen AC, et al. The effect of providing prescribing recommendations on appropriate prescribing: A cluster-randomized controlled trial in older adults in a preoperative setting. Br J Clin Pharmacol. 2019 Sep;85(9):1974-83. doi: 10.1111/bcp.13987. PMID: 31108564. *Design*

9. Boyd CM, Shetterly SM, Powers JD, et al. Evaluating the Safety of an Educational Deprescribing Intervention: Lessons from the Optimize Trial. Drugs Aging. 2024 Jan;41(1):45-54. doi: 10.1007/s40266-023-01080-y. PMID: 37982982. *Outcome*

10. Brokaar EJ, van den Bos F, Visser LE, et al. Deprescribing in Older Adults With Cancer and Limited Life Expectancy: An Integrative Review. Am J Hosp Palliat Care. 2022 Jan;39(1):86-100. doi: 10.1177/10499091211003078. PMID: 33739162. *Intervention*

11. Campbell NL, Perkins AJ, Khan BA, et al. Deprescribing in the Pharmacologic Management of Delirium: A Randomized Trial in the Intensive Care Unit. J Am Geriatr Soc. 2019 Apr;67(4):695-702. doi: 10.1111/jgs.15751. PMID: 30664239. *Setting*

12. Cateau D, Ballabeni P, Mena S, et al. Deprescribing in nursing homes: Protocol for nested, randomised controlled hybrid trials of deprescribing interventions. Res Social Adm Pharm. 2021 Apr;17(4):786-94. doi: 10.1016/j.sapharm.2020.05.026. PMID: 32622774. *Setting*

© 2025 Linsky AM et al. *JAMA Network Open*.

13. Cateau D, Ballabeni P, Niquille A. Effects of an interprofessional deprescribing intervention in Swiss nursing homes: the Individual Deprescribing Intervention (IDeI) randomised controlled trial. BMC Geriatr. 2021 Nov 19;21(1):655. doi: 10.1186/s12877-021-02465-7. PMID: 34798826. *Setting*

14. Cateau D, Ballabeni P, Niquille A. Effects of an interprofessional Quality Circle-Deprescribing Module (QC-DeMo) in Swiss nursing homes: a randomised controlled trial. BMC Geriatr. 2021 May 1;21(1):289. doi: 10.1186/s12877-021-02220-y. PMID: 33933030. *Setting*

15. Christopher CM, Kc B, Blebil A, et al. Clinical and Humanistic Outcomes of Community Pharmacy-Based Healthcare Interventions Regarding Medication Use in Older Adults: A Systematic Review and Meta-Analysis. Healthcare (Basel). 2021 Nov 18;9(11). doi: 10.3390/healthcare9111577. PMID: 34828622. *Intervention*

16. Cicco S, M DA, Proietti M, et al. Antihypertensive treatment changes and related clinical outcomes in older hospitalized patients. Eur J Clin Invest. 2023 Apr;53(4):e13931. doi: 10.1111/eci.13931. PMID: 36453932. *Design*

17. Clarkson L, Hart L, Lam AK, et al. Reducing inappropriate polypharmacy for older patients at specialist outpatient clinics: a systematic review. Curr Med Res Opin. 2023 Apr;39(4):545-54. doi: 10.1080/03007995.2023.2185390. PMID: 36847597. *Not on topic*

18. Cochrane Special Collections. Achieving sustainable healthcare through deprescribing of unnecessary medications: making sense of the evidence. Cochrane Library. 2022. doi: SC000054. *Design*

19. Crotty M, Whitehead C, Rowett D, et al. An outreach intervention to implement evidence based practice in residential care: a randomized controlled trial [ISRCTN67855475]. BMC Health Serv Res. 2004 Apr 6;4(1):6. doi: 10.1186/1472-6963-4-6. PMID: 15066200. *Setting*

20. Crutzen S, Baas G, Denig P, et al. Pharmacist-led intervention aimed at deprescribing and appropriate use of cardiometabolic medication among people with type 2 diabetes. Res Social Adm Pharm. 2023 May;19(5):783-92. doi: 10.1016/j.sapharm.2022.11.009. PMID: 36740525. *Design*

21. Curtin D, Jennings E, Daunt R, et al. Deprescribing in Older People Approaching End of Life: A Randomized Controlled Trial Using STOPPFrail Criteria. J Am Geriatr Soc. 2020 Apr;68(4):762-9. doi: 10.1111/jgs.16278. PMID: 31868920. *Setting*

22. Dagnew SB, Binega Mekonnen G, Gebeye Zeleke E, et al. Clinical Pharmacist Intervention on Drug-Related Problems among Elderly Patients Admitted to Medical Wards of Northwest Ethiopia Comprehensive Specialized Hospitals: A Multicenter Prospective, Observational Study. Biomed Res Int. 2022;2022:8742998. doi: 10.1155/2022/8742998. PMID: 35898673. *Design*

23. Debacq C, Bourgueil J, Aidoud A, et al. Persistence of Effect of Medication Review on Potentially Inappropriate Prescriptions in Older Patients Following Hospital Discharge. Drugs Aging. 2021 Mar;38(3):243-52. doi: 10.1007/s40266-020-00830-6. PMID: 33474671. *Design*

24. Dellinger JK, Pitzer S, Schaffler-Schaden D, et al. Improving medication appropriateness in nursing homes via structured interprofessional medication-review supported by health information technology: a non-randomized controlled study. BMC Geriatr. 2020 Nov 26;20(1):506. doi: 10.1186/s12877-020-01895-z. PMID: 33243145. *Design*

© 2025 Linsky AM et al. *JAMA Network Open*.

25. Deng Z, Thompson W, Korenvain C, et al. Benefits and Harms of Deprescribing Antihyperglycemics for Adults With Type 2 Diabetes: A Systematic Review. Can J Diabetes. 2022 Jul;46(5):473-9. doi: 10.1016/j.jcjd.2022.01.009. PMID: 35739041. *Intervention*

26. Eastern AHSN. Opioid deprescribing toolkit. n.d. https://easternahsn.org/about-us/our-projects/opioid-deprescribing-toolkit/. Accessed on September 22, 2023. *Toolkit*

27. Elbeddini A, Sawhney M, Tayefehchamani Y, et al. Deprescribing for all: a narrative review identifying inappropriate polypharmacy for all ages in hospital settings. BMJ Open Qual. 2021 Jul;10(3). doi: 10.1136/bmjoq-2021-001509. PMID: 34230053. *Design*

28. Etherton-Beer C, Page A, Naganathan V, et al. Deprescribing to optimise health outcomes for frail older people: a double-blind placebo-controlled randomised controlled trial-outcomes of the Opti-med study. Age Ageing. 2023 May 1;52(5). doi: 10.1093/ageing/afad081. PMID: 37247404. *Setting*

29. Evrard P, Henrard S, Foulon V, et al. Benzodiazepine Use and Deprescribing in Belgian Nursing Homes: Results from the COME-ON Study. J Am Geriatr Soc. 2020 Dec;68(12):2768-77. doi: 10.1111/jgs.16751. PMID: 32786002. *Setting*

30. Farhat A, Al-Hajje A, Lang PO, et al. Impact of Pharmaceutical Interventions with STOPP/START and PIM-Check in Older Hospitalized Patients: A Randomized Controlled Trial. Drugs Aging. 2022 Nov;39(11):899-910. doi: 10.1007/s40266-022-00974-7. PMID: 36175740. *Intervention*

31. Fournier A, Anrys P, Beuscart JB, et al. Use and Deprescribing of Potentially Inappropriate Medications in Frail Nursing Home Residents. Drugs Aging. 2020 Dec;37(12):917-24. doi: 10.1007/s40266-020-00805-7. PMID: 33047252. *Setting*

32. Gedde MH, Husebo BS, Mannseth J, et al. Less Is More: The Impact of Deprescribing Psychotropic Drugs on Behavioral and Psychological Symptoms and Daily Functioning in Nursing Home Patients. Results From the Cluster-Randomized Controlled COSMOS Trial. Am J Geriatr Psychiatry. 2021 Mar;29(3):304-15. doi: 10.1016/j.jagp.2020.07.004. PMID: 32753339. *Setting*

33. GeriatricsCareOnline.org. AGS Deprescribing Toolkit. n.d. https://geriatricscareonline.org/toc/ags-deprescribing-toolkit/TK013. Accessed on September 22, 2023. *Toolkit*

34. Groot Kormelinck CM, van Teunenbroek CF, Zuidema SU, et al. Process evaluation of a tailored intervention to Reduce Inappropriate psychotropic Drug use in nursing home residents with dementia. BMC Geriatr. 2021 Jul 3;21(1):414. doi: 10.1186/s12877-021-02357-w. PMID: 34217230. *Outcome*

35. Ha M, Furman A, Al Rihani SB, et al. Pharmacist-driven interventions to de-escalate urinary antimuscarinics in the Programs of All-Inclusive Care for the Elderly. J Am Geriatr Soc. 2022 Nov;70(11):3230-8. doi: 10.1111/jgs.17965. PMID: 35900034. *Design*

36. Hickman E, Seawoodharry M, Gillies C, et al. Deprescribing in cardiometabolic conditions in older patients: a systematic review. Geroscience. 2023 Jul 5. doi: 10.1007/s11357-023-00852-z. PMID: 37402905. *Intervention*

© 2025 Linsky AM et al. *JAMA Network Open*.

37. Hirdes JP, Major J, Didic S, et al. A Canadian Cohort Study to Evaluate the Outcomes Associated with a Multicenter Initiative to Reduce Antipsychotic Use in Long-Term Care Homes. J Am Med Dir Assoc. 2020 Jun;21(6):817-22. doi: 10.1016/j.jamda.2020.04.004. PMID: 32493650. *Design*

38. Jungo KT, Ansorg AK, Floriani C, et al. Optimising prescribing in older adults with multimorbidity and polypharmacy in primary care (OPTICA): cluster randomised clinical trial. Bmj. 2023 May 24;381:e074054. doi: 10.1136/bmj-2022-074054. PMID: 37225248. *Not on topic*

39. Junius-Walker U, Krause O, Thürmann P, et al. Drug Safety for Nursing-Home Residents-Findings of a Pragmatic, Cluster-Randomized, Controlled Intervention Trialin 44 Nursing Homes. Dtsch Arztebl Int. 2021 Oct 22;118(42):705-12. doi: 10.3238/arztebl.m2021.0297. PMID: 34366004. *Intervention*

40. Juraschek SP, Cluett JL, Belanger MJ, et al. Effects of Antihypertensive Deprescribing Strategies on Blood Pressure, Adverse Events, and Orthostatic Symptoms in Older Adults: Results From TONE. Am J Hypertens. 2022 Apr 2;35(4):337-46. doi: 10.1093/ajh/hpab171. PMID: 34718403. *Intervention*

41. Kaminaga M, Komagamine J, Tatsumi S. The effects of in-hospital deprescribing on potential prescribing omission in hospitalized elderly patients with polypharmacy. Sci Rep. 2021 Apr 26;11(1):8898. doi: 10.1038/s41598-021-88362-w. PMID: 33903645. *Design*

42. Kim JL, Lewallen KM, Hollingsworth EK, et al. Patient-Reported Barriers and Enablers to Deprescribing Recommendations During a Clinical Trial (Shed-MEDS). Gerontologist. 2023 Mar 21;63(3):523-33. doi: 10.1093/geront/gnac100. PMID: 35881109. *Outcome*

43. Kornholt J, Feizi ST, Hansen AS, et al. Medication changes implemented during medication reviews and factors related to deprescribing: Posthoc analyses of a randomized clinical trial in geriatric outpatients with polypharmacy. Br J Clin Pharmacol. 2023 Nov;89(11):3291-301. doi: 10.1111/bcp.15805. PMID: 37254818. *Setting*

44. Kose E, Endo H, Hori H, et al. Association of Pharmacist-led Deprescribing Intervention with the Functional Recovery in Convalescent Setting. Pharmazie. 2022 May 1;77(5):165-70. doi: 10.1691/ph.2022.2323. PMID: 35655381. *Design*

45. Kua CH, Yeo CYY, Tan PC, et al. Association of Deprescribing With Reduction in Mortality and Hospitalization: A Pragmatic Stepped-Wedge Cluster-Randomized Controlled Trial. J Am Med Dir Assoc. 2021 Jan;22(1):82-9.e3. doi: 10.1016/j.jamda.2020.03.012. PMID: 32423694. *Setting*

46. Lamarre M, Marcotte M, Laurin D, et al. Discontinuation of bisphosphonates in seniors: a systematic review on health outcomes. Arch Osteoporos. 2021 Sep 15;16(1):133. doi: 10.1007/s11657-021-01000-w. PMID: 34524561. *Intervention*

47. Lee JW, Boyd CM, Leff B, et al. Tailoring a home-based, multidisciplinary deprescribing intervention through clinicians and community-dwelling older adults. J Am Geriatr Soc. 2023 May;71(5):1663-6. doi: 10.1111/jgs.18186. PMID: 36515689. *Design*

48. Maust DT, Takamine L, Wiechers IR, et al. Strategies Associated With Reducing Benzodiazepine Prescribing to Older Adults: A Mixed Methods Study. Ann Fam Med. 2022 Jul-Aug;20(4):328-35. doi: 10.1370/afm.2825. PMID: 35879067. *Outcome*

© 2025 Linsky AM et al. *JAMA Network Open*.

49. McDonald EG, Wu PE, Rashidi B, et al. The MedSafer Study-Electronic Decision Support for Deprescribing in Hospitalized Older Adults: A Cluster Randomized Clinical Trial. JAMA Intern Med. 2022 Mar 1;182(3):265-73. doi: 10.1001/jamainternmed.2021.7429. PMID: 35040926. *Setting*

50. Mozer C, Madden W, Cursio J, et al. Rates of Inappropriate Dosing in Older Adults at an Urban, Academic Hospital. Am J Med Qual. 2021 Nov-Dec 01;36(6):469-70. doi: 10.1097/01.Jmq.0000751156.52061.64. PMID: 34038914. *Design*

51. Mucherino S, Casula M, Galimberti F, et al. The Effectiveness of Interventions to Evaluate and Reduce Healthcare Costs of Potentially Inappropriate Prescriptions among the Older Adults: A Systematic Review. Int J Environ Res Public Health. 2022 May 31;19(11). doi: 10.3390/ijerph19116724. PMID: 35682331. *Not on topic*

52. National Hospice and Palliative Care Organization. Hospice Medication Deprescribing Toolkit. November 2020. https://www.nhpco.org/wp-content/uploads/NHPCO_Deprescribing_Toolkit.pdf. *Toolkit*

53. O'Mahony D, Gudmundsson A, Soiza RL, et al. Prevention of adverse drug reactions in hospitalized older patients with multi-morbidity and polypharmacy: the SENATOR* randomized controlled clinical trial. Age Ageing. 2020 Jul 1;49(4):605-14. doi: 10.1093/ageing/afaa072. PMID: 32484850. *Setting*

54. Pavon JM, Davidson S, Sloane R, et al. Deprescribing electronic case reviews for older veterans at risk for falls: Effects on drug burden and falls. J Am Geriatr Soc. 2024 Feb;72(2):433-43. doi: 10.1111/jgs.18650. PMID: 37941488. *Design*

55. Persell SD, Brown T, Doctor JN, et al. Development of High-Risk Geriatric Polypharmacy Electronic Clinical Quality Measures and a Pilot Test of EHR Nudges Based on These Measures. J Gen Intern Med. 2022 Aug;37(11):2777-85. doi: 10.1007/s11606-021-07296-1. PMID: 34993860. *Not on topic*

56. Pruskowski JA, Springer S, Thorpe CT, et al. Does Deprescribing Improve Quality of Life? A Systematic Review of the Literature. Drugs Aging. 2019 Dec;36(12):1097-110. doi: 10.1007/s40266-019-00717-1. PMID: 31598908. *Intervention*

57. Quek HW, Etherton-Beer C, Page A, et al. Deprescribing for older people living in residential aged care facilities: Pharmacist recommendations, doctor acceptance and implementation. Arch Gerontol Geriatr. 2023 Apr;107:104910. doi: 10.1016/j.archger.2022.104910. PMID: 36565605. *Setting*

58. Rantsi M, Pitkälä KH, Kautiainen H, et al. Cost-effectiveness of an educational intervention to reduce potentially inappropriate medication. Age Ageing. 2022 May 1;51(5). doi: 10.1093/ageing/afac112. PMID: 35604803. *Design*

59. Rashid R, Chang C, Niu F, et al. Evaluation of a Pharmacist-Managed Nonsteroidal Anti-Inflammatory Drugs Deprescribing Program in an Integrated Health Care System. J Manag Care Spec Pharm. 2020 Jul;26(7):918-24. doi: 10.18553/jmcp.2020.26.7.918. PMID: 32584681. *Design*

60. Reeve E, Jordan V, Thompson W, et al. Withdrawal of antihypertensive drugs in older people. Cochrane Database Syst Rev. 2020 Jun 10;6(6):Cd012572. doi: 10.1002/14651858.CD012572.pub2. PMID: 32519776. *Intervention*

© 2025 Linsky AM et al. *JAMA Network Open*.

61. Sacarny A, Barnett ML, Le J, et al. Effect of Peer Comparison Letters for High-Volume Primary Care Prescribers of Quetiapine in Older and Disabled Adults: A Randomized Clinical Trial. JAMA Psychiatry. 2018 Oct 1;75(10):1003-11. doi: 10.1001/jamapsychiatry.2018.1867. PMID: 30073273. *Outcome*

62. Sanyal C, Turner JP, Martin P, et al. Cost-Effectiveness of Pharmacist-Led Deprescribing of NSAIDs in Community-Dwelling Older Adults. J Am Geriatr Soc. 2020 May;68(5):1090-7. doi: 10.1111/jgs.16388. PMID: 32105355. *Outcome*

63. Sawan MJ, Moga DC, Ma MJ, et al. The value of deprescribing in older adults with dementia: a narrative review. Expert Rev Clin Pharmacol. 2021 Nov;14(11):1367-82. doi: 10.1080/17512433.2021.1961576. PMID: 34311630. *Design*

64. Seidu S, Kunutsor SK, Topsever P, et al. Deintensification in older patients with type 2 diabetes: A systematic review of approaches, rates and outcomes. Diabetes Obes Metab. 2019 Jul;21(7):1668-79. doi: 10.1111/dom.13724. PMID: 30938038. *Intervention*

65. Seto H, Ishimaru N, Ohnishi J, et al. Multidisciplinary Team Deprescribing Intervention for Polypharmacy in Elderly Orthopedic Inpatients: A Propensity Score-matched Analysis of a Retrospective Cohort Study. Intern Med. 2022 Aug 15;61(16):2417-26. doi: 10.2169/internalmedicine.8929-21. PMID: 35022357. *Design*

66. Sheehan OC, Gleason KS, Bayliss EA, et al. Intervention design in cognitively impaired populations-Lessons learned from the OPTIMIZE deprescribing pragmatic trial. J Am Geriatr Soc. 2023 Mar;71(3):774-84. doi: 10.1111/jgs.18148. PMID: 36508725. *Outcome*

67. Sheppard JP, Burt J, Lown M, et al. Effect of Antihypertensive Medication Reduction vs Usual Care on Short-term Blood Pressure Control in Patients With Hypertension Aged 80 Years and Older: The OPTIMISE Randomized Clinical Trial. Jama. 2020 May 26;323(20):2039-51. doi: 10.1001/jama.2020.4871. PMID: 32453368. *Not on topic*

68. Shrestha S, Poudel A, Forough AS, et al. A systematic review on methods for developing and validating deprescribing tools for older adults with limited life expectancy. Int J Pharm Pract. 2023 Mar 13;31(1):3-14. doi: 10.1093/ijpp/riac094. PMID: 36472946. *Intervention*

69. Stötzner P, Ferrebus Abate RE, Henssler J, et al. Structured Interventions to Optimize Polypharmacy in Psychiatric Treatment and Nursing Homes: A Systematic Review. J Clin Psychopharmacol. 2022 Mar-Apr 01;42(2):169-87. doi: 10.1097/jcp.0000000000001521. PMID: 35230048. *Not on topic*

70. Strauven G, Anrys P, Vandael E, et al. Cluster-Controlled Trial of an Intervention to Improve Prescribing in Nursing Homes Study. J Am Med Dir Assoc. 2019 Nov;20(11):1404-11. doi: 10.1016/j.jamda.2019.06.006. PMID: 31402136. *Setting*

71. Tadrous M, Fung K, Desveaux L, et al. Effect of Academic Detailing on Promoting Appropriate Prescribing of Antipsychotic Medication in Nursing Homes: A Cluster Randomized Clinical Trial. JAMA Netw Open. 2020 May 1;3(5):e205724. doi: 10.1001/jamanetworkopen.2020.5724. PMID: 32453383. *Setting*

72. Taylor-Rowan M, Alharthi AA, Noel-Storr AH, et al. Anticholinergic deprescribing interventions for reducing risk of cognitive decline or dementia in older adults with and without prior cognitive impairment. Cochrane Database of Systematic Reviews. 2022(12). doi: 10.1002/14651858.CD015405. PMID: CD015405. *Not on topic*

© 2025 Linsky AM et al. *JAMA Network Open*.

73. Taylor-Rowan M, Alharthi AA, Noel-Storr AH, et al. Anticholinergic deprescribing interventions for reducing risk of cognitive decline or dementia in older adults with and without prior cognitive impairment. Cochrane Database of Systematic Reviews. 2023(12). doi: 10.1002/14651858.CD015405.pub2. PMID: CD015405. *Outcome*

74. Thompson W, Lundby C, Graabaek T, et al. Tools for Deprescribing in Frail Older Persons and Those with Limited Life Expectancy: A Systematic Review. J Am Geriatr Soc. 2019 Jan;67(1):172-80. doi: 10.1111/jgs.15616. PMID: 30315745. *Outcome*

75. Turner JP, Sanyal C, Martin P, et al. Economic Evaluation of Sedative Deprescribing in Older Adults by Community Pharmacists. J Gerontol A Biol Sci Med Sci. 2021 May 22;76(6):1061-7. doi: 10.1093/gerona/glaa180. PMID: 32761069. *Outcome*

76. Ulley J, Harrop D, Ali A, et al. Deprescribing interventions and their impact on medication adherence in community-dwelling older adults with polypharmacy: a systematic review. BMC Geriatr. 2019 Jan 18;19(1):15. doi: 10.1186/s12877-019-1031-4. PMID: 30658576. *Not on topic*

77. van der Worp H, Jellema P, Hordijk I, et al. Discontinuation of alpha-blocker therapy in men with lower urinary tract symptoms: a systematic review and meta-analysis. BMJ Open. 2019 Nov 7;9(11):e030405. doi: 10.1136/bmjopen-2019-030405. PMID: 31699724. *Intervention*

78. Vasilevskis EE, Shah AS, Hollingsworth EK, et al. Deprescribing Medications Among Older Adults From End of Hospitalization Through Postacute Care: A Shed-MEDS Randomized Clinical Trial. JAMA Intern Med. 2023 Mar 1;183(3):223-31. doi: 10.1001/jamainternmed.2022.6545. PMID: 36745422. *Setting*

79. Vaughan CP, Burningham Z, Kelleher JL, et al. A cluster-randomized trial of two implementation strategies to deliver audit and feedback in the EQUIPPED medication safety program. Acad Emerg Med. 2023 Apr;30(4):340-8. doi: 10.1111/acem.14697. PMID: 36790188. *Not on topic*

80. Wong APY, Ting TW, Charissa EJM, et al. Feasibility & Efficacy of Deprescribing rounds in a Singapore rehabilitative hospital- a randomised controlled trial. BMC Geriatr. 2021 Oct 21;21(1):584. doi: 10.1186/s12877-021-02507-0. PMID: 34674645. *Setting*

Background studies

1. Cole JA, Gonçalves-Bradley DC, Alqahtani M, et al. Interventions to improve the appropriate use of polypharmacy for older people. Cochrane Database of Systematic Reviews. 2023(10). doi: 10.1002/14651858.CD008165.pub5. PMID: CD008165. *Background*

2. O'Mahony D. STOPP/START criteria for potentially inappropriate medications/potential prescribing omissions in older people: origin and progress. Expert Rev Clin Pharmacol. 2020 Jan;13(1):15-22. doi: 10.1080/17512433.2020.1697676. PMID: 31790317. *Background*

3. Reeve E, To J, Hendrix I, et al. Patient barriers to and enablers of deprescribing: a systematic review. Drugs Aging. 2013 Oct;30(10):793-807. doi: 10.1007/s40266-013-0106-8. PMID: 23912674. *Background*

4. Reeve J, Maden M, Hill R, et al. Deprescribing medicines in older people living with multimorbidity and polypharmacy: the TAILOR evidence synthesis. Health Technol Assess. 2022 Jul;26(32):1-148. doi: 10.3310/aafo2475. PMID: 35894932. *Background*

© 2025 Linsky AM et al. *JAMA Network Open*.

5. Saeed D, Carter G, Parsons C. Interventions to improve medicines optimisation in frail older patients in secondary and acute care settings: a systematic review of randomised controlled trials and non-randomised studies. Int J Clin Pharm. 2022 Feb;44(1):15-26. doi: 10.1007/s11096-021-01354-8. PMID: 34800255. *Background*

6. Santos NSD, Marengo LL, Moraes FDS, et al. Interventions to reduce the prescription of inappropriate medicines in older patients. Rev Saude Publica. 2019 Jan 31;53:7. doi: 10.11606/s1518-8787.2019053000781. PMID: 30726488. *Background*

7. Steinman MA, Boyd CM, Spar MJ, et al. Deprescribing and deimplementation: Time for transformative change. J Am Geriatr Soc. 2021 Dec;69(12):3693-5. doi: 10.1111/jgs.17441. PMID: 34499742. *Background*

8. Veronese N, Gallo U, Boccardi V, et al. Efficacy of deprescribing on health outcomes: An umbrella review of systematic reviews with meta-analysis of randomized controlled trials. Ageing Res Rev. 2024 Mar;95:102237. doi: 10.1016/j.arr.2024.102237. PMID: 38367812. *Background*

Systematic Reviews

1. Ali MU, Sherifali D, Fitzpatrick-Lewis D, et al. Interventions to address polypharmacy in older adults living with multimorbidity: Review of reviews. Can Fam Physician. 2022 Jul;68(7):e215-e26. doi: 10.46747/cfp.6807e215. PMID: 35831093. *Systematic Review*

2. Bloomfield H, Linsky A, Bolduc J, et al. VA Evidence-based Synthesis Program Reports. Deprescribing for Older Veterans: A Systematic Review. Washington (DC): Department of Veterans Affairs (US); 2019. *Systematic Review*

3. Bloomfield HE, Greer N, Linsky AM, et al. Deprescribing for Community-Dwelling Older Adults: a Systematic Review and Meta-analysis. J Gen Intern Med. 2020 Nov;35(11):3323-32. doi: 10.1007/s11606-020-06089-2. PMID: 32820421. *Systematic Review*

4. Brunner L, Rodondi N, Aubert CE. Barriers and facilitators to deprescribing of cardiovascular medications: a systematic review. BMJ Open. 2022 Dec 22;12(12):e061686. doi: 10.1136/bmjopen-2022-061686. PMID: 36549739. *Systematic Review*

5. Buzancic I, Kummer I, Drzaic M, et al. Community-based pharmacists' role in deprescribing: A systematic review. Br J Clin Pharmacol. 2022 Feb;88(2):452-63. doi: 10.1111/bcp.14947. PMID: 34155673. *Systematic Review*

6. Cardona M, Stehlik P, Fawzy P, et al. Effectiveness and sustainability of deprescribing for hospitalized older patients near end of life: a systematic review. Expert Opin Drug Saf. 2021 Jan;20(1):81-91. doi: 10.1080/14740338.2021.1853704. PMID: 33213216. *Systematic Review*

7. Crisafulli S, Luxi N, Coppini R, et al. Anti-hypertensive drugs deprescribing: an updated systematic review of clinical trials. BMC Fam Pract. 2021 Oct 20;22(1):208. doi: 10.1186/s12875-021-01557-y. PMID: 34666689. *Systematic Review*

8. Doherty AJ, Boland P, Reed J, et al. Barriers and facilitators to deprescribing in primary care: a systematic review. BJGP Open. 2020 Aug;4(3). doi: 10.3399/bjgpopen20X101096. PMID: 32723784. *Systematic Review*

© 2025 Linsky AM et al. *JAMA Network Open*.

9. Earl TR, Katapodis ND, Schneiderman SR, et al. Using Deprescribing Practices and the Screening Tool of Older Persons' Potentially Inappropriate Prescriptions Criteria to Reduce Harm and Preventable Adverse Drug Events in Older Adults. J Patient Saf. 2020 Sep;16(3S Suppl 1):S23-s35. doi: 10.1097/pts.0000000000000747. PMID: 32809998. *Systematic Review*

10. Ibrahim K, Cox NJ, Stevenson JM, et al. A systematic review of the evidence for deprescribing interventions among older people living with frailty. BMC Geriatr. 2021 Apr 17;21(1):258. doi: 10.1186/s12877-021-02208-8. PMID: 33865310. *Systematic Review*

11. Kua CH, Mak VSL, Huey Lee SW. Health Outcomes of Deprescribing Interventions Among Older Residents in Nursing Homes: A Systematic Review and Meta-analysis. J Am Med Dir Assoc. 2019 Mar;20(3):362-72.e11. doi: 10.1016/j.jamda.2018.10.026. PMID: 30581126. *Systematic Review*

12. Lee J, Negm A, Peters R, et al. Deprescribing fall-risk increasing drugs (FRIDs) for the prevention of falls and fall-related complications: a systematic review and meta-analysis. BMJ Open. 2021 Feb 10;11(2):e035978. doi: 10.1136/bmjopen-2019-035978. PMID: 33568364. *Systematic Review*

13. Lee JW, Li M, Boyd CM, et al. Preoperative Deprescribing for Medical Optimization of Older Adults Undergoing Surgery: A Systematic Review. J Am Med Dir Assoc. 2022 Apr;23(4):528-36.e2. doi: 10.1016/j.jamda.2021.11.005. PMID: 34861224. *Systematic Review*

14. Monteiro L, Maricoto T, Solha I, et al. Reducing Potentially Inappropriate Prescriptions for Older Patients Using Computerized Decision Support Tools: Systematic Review. J Med Internet Res. 2019 Nov 14;21(11):e15385. doi: 10.2196/15385. PMID: 31724956. *Systematic Review*

15. Nguyen M, Beier MT, Louden DN, et al. The Effect of Pharmacist-Initiated Deprescribing Interventions in Older People: A Narrative Review of Randomized Controlled Trials. Sr Care Pharm. 2023 Dec 1;38(12):506-23. doi: 10.4140/TCP.n.2023.506. PMID: 38041222. *Systematic Review*

16. Niznik JD, Collins BJ, Armistead LT, et al. Pharmacist interventions to deprescribe opioids and benzodiazepines in older adults: A rapid review. Res Social Adm Pharm. 2022 Jun;18(6):2913-21. doi: 10.1016/j.sapharm.2021.07.012. PMID: 34281786. *Systematic Review*

17. Okeowo DA, Zaidi STR, Fylan B, et al. Barriers and facilitators of implementing proactive deprescribing within primary care: a systematic review. Int J Pharm Pract. 2023 Apr 10;31(2):126-52. doi: 10.1093/ijpp/riad001. PMID: 36860190. *Systematic Review*

18. Omuya H, Nickel C, Wilson P, et al. A systematic review of randomised-controlled trials on deprescribing outcomes in older adults with polypharmacy. Int J Pharm Pract. 2023 Jun 30;31(4):349-68. doi: 10.1093/ijpp/riad025. PMID: 37155330. *Systematic Review*

19. Ribeiro PRS, Schlindwein AD. Benzodiazepine deprescription strategies in chronic users: a systematic review. Fam Pract. 2021 Sep 25;38(5):684-93. doi: 10.1093/fampra/cmab017. PMID: 33907803. *Systematic Review*

20. Rodrigues DA, Plácido AI, Mateos-Campos R, et al. Effectiveness of Interventions to Reduce Potentially Inappropriate Medication in Older Patients: A Systematic Review. Front Pharmacol. 2021;12:777655. doi: 10.3389/fphar.2021.777655. PMID: 35140603. *Systematic Review*

© 2025 Linsky AM et al. *JAMA Network Open*.

21. Romano S, Figueira D, Teixeira I, et al. Deprescribing Interventions among Community-Dwelling Older Adults: A Systematic Review of Economic Evaluations. Pharmacoeconomics. 2022 Mar;40(3):269-95. doi: 10.1007/s40273-021-01120-8. PMID: 34913143. *Systematic Review*

22. Salahudeen MS, Alfahmi A, Farooq A, et al. Effectiveness of Interventions to Improve the Anticholinergic Prescribing Practice in Older Adults: A Systematic Review. J Clin Med. 2022 Jan 28;11(3). doi: 10.3390/jcm11030714. PMID: 35160166. *Systematic Review*

23. Seppala LJ, Kamkar N, van Poelgeest EP, et al. Medication reviews and deprescribing as a single intervention in falls prevention: a systematic review and meta-analysis. Age Ageing. 2022 Sep 2;51(9). doi: 10.1093/ageing/afac191. PMID: 36153749. *Systematic Review*

24. Shrestha S, Poudel A, Cardona M, et al. Impact of deprescribing dual-purpose medications on patient-related outcomes for older adults near end-of-life: a systematic review and meta-analysis. Ther Adv Drug Saf. 2021;12:20420986211052343. doi: 10.1177/20420986211052343. PMID: 34707802. *Systematic Review*

25. Shrestha S, Poudel A, Steadman K, et al. Outcomes of deprescribing interventions in older patients with life-limiting illness and limited life expectancy: A systematic review. Br J Clin Pharmacol. 2020 Oct;86(10):1931-45. doi: 10.1111/bcp.14113. PMID: 31483057. *Systematic Review*

26. Sirois C, Gosselin M, Laforce C, et al. How does deprescribing (not) reduce mortality? A review of a meta-analysis in community-dwelling older adults casts uncertainty over claimed benefits. Basic Clin Pharmacol Toxicol. 2023 Jun 27. doi: 10.1111/bcpt.13921. PMID: 37376746. *Systematic Review*

27. Turk A, Wong G, Mahtani KR, et al. Optimising a person-centred approach to stopping medicines in older people with multimorbidity and polypharmacy using the DExTruS framework: a realist review. BMC Med. 2022 Aug 31;20(1):297. doi: 10.1186/s12916-022-02475-1. PMID: 36042454. *Systematic Review*

© 2025 Linsky AM et al. *JAMA Network Open*.

Supplemental File 3. Cochrane Risk of Bias for RCTs

eTable 1. Cochrane Risk of Bias for RCTs

Author, Year	Random	Allocation Concealment	Blinding Participants	Blinding Outcome Assessment	Selective Reporting	Attrition
Amorim, 2024[1]	Low Risk	Uncertain Risk	Low Risk	Low Risk	Low Risk	Low Risk
Bayliss, 2022[2]	Low Risk	Low Risk	High Risk	Low Risk	Low Risk	Low Risk
Campbell, 2021[3]	Low Risk	Uncertain Risk	High Risk	Low Risk	Low Risk	High Risk
Harnisch, 2024[4]	Low Risk	Low Risk	Low Risk	Low Risk	Low Risk	Low Risk
Herrinton, 2023[5]	Low Risk	Low Risk	High Risk	Low risk	Low Risk	Low Risk
Jamieson, 2023[6]	Low Risk	Low Risk	Uncertain Risk	Uncertain Risk	Low Risk	Low Risk
Kouladjian, 2021[7]	Low Risk	Uncertain Risk	High Risk	High Risk	Low Risk	Uncertain Risk
Kuntz, 2019[8]	Low Risk	Uncertain Risk	High Risk	Low Risk	Low Risk	Low Risk
Mahlknecht, 2021[9]	Low Risk	Low Risk	High Risk	High Risk	Low Risk	Low Risk
Mak, 2022[10]	Low Risk	Low Risk	Low Risk	Low Risk	Low Risk	Low Risk
McCarthy, 2022[11]	Low Risk	Low Risk	High Risk	High Risk	Low Risk	Low Risk
Mortsiefer, 2023[12]	Low Risk	Low Risk	High Risk	High Risk	Low Risk	Uncertain Risk
Phelan, 2024[13]	Low Risk	Low Risk	Uncertain Risk	Low Risk	Low Risk	Low Risk
Rieckert, 2020[14]	Low Risk	Low Risk	High Risk	Low Risk	Low Risk	Uncertain Risk
Rudolf, 2021[15]	Low Risk	Low Risk	Uncertain Risk	High Risk	Low Risk	Uncertain Risk
Wallis, 2022[16]	Low Risk	Low Risk	High Risk	Low Risk	Low Risk	Uncertain Risk
Zechmann, 2020[17]	Low Risk	Uncertain Risk	Uncertain Risk	Uncertain Risk	Low Risk	Low Risk

Note: For attrition, 80% followup used to assign low risk; 70%–80% = uncertain risk, <70% = high risk

© 2025 Linsky AM et al. *JAMA Network Open*.

eTable 2. GRADE Certainty of Evidence Table

Intervention	Study limitations	Consistency	Directness	Precision	Certainty of evidence
Deprescribing interventions to reduce polypharmacy	No serious limitations	Serious inconsistency	No serious indirectness	No serious imprecision	Moderate

© 2025 Linsky AM et al. *JAMA Network Open*.

Supplemental File References

1. Amorim WW, Passos LC, Gama RS, et al. Using a mobile application to reduce potentially inappropriate prescribing for older Brazilian adults in primary care: a triple-blind randomised clinical trial. *BMC Geriatr.* 2024;24(1):35.
2. Bayliss EA, Shetterly SM, Drace ML, et al. Deprescribing Education vs Usual Care for Patients With Cognitive Impairment and Primary Care Clinicians: The OPTIMIZE Pragmatic Cluster Randomized Trial. *JAMA Intern Med.* 2022;182(5):534-542.
3. Campbell NL, Holden RJ, Tang Q, et al. Multicomponent behavioral intervention to reduce exposure to anticholinergics in primary care older adults. *J Am Geriatr Soc.* 2021;69(6):1490-1499.
4. Harnisch M, Barnett ML, Coussens S, et al. Physician Antipsychotic Overprescribing Letters and Cognitive, Behavioral, and Physical Health Outcomes Among People With Dementia: A Secondary Analysis of a Randomized Clinical Trial. *JAMA Netw Open.* 2024;7(4):e247604.
5. Herrinton LJ, Lo K, Alavi M, et al. Effectiveness of Bundled Hyperpolypharmacy Deprescribing Compared With Usual Care Among Older Adults: A Randomized Clinical Trial. *JAMA Netw Open.* 2023;6(7):e2322505.
6. Jamieson H, Nishtala PS, Bergler HU, et al. Deprescribing anticholinergic and sedative drugs to reduce polypharmacy in frail older adults living in the community: a randomized controlled trial. *J Gerontol A Biol Sci Med Sci.* 2023.
7. Kouladjian O'Donnell L, Gnjidic D, Sawan M, et al. Impact of the Goal-directed Medication Review Electronic Decision Support System on Drug Burden Index: A cluster-randomised clinical trial in primary care. *Br J Clin Pharmacol.* 2021;87(3):1499-1511.
8. Kuntz JL, Kouch L, Christian D, Hu W, Peterson PL. Patient Education and Pharmacist Consultation Influence on Nonbenzodiazepine Sedative Medication Deprescribing Success for Older Adults. *Perm J.* 2019;23:18-161.
9. Mahlknecht A, Wiedermann CJ, Sandri M, et al. Expert-based medication reviews to reduce polypharmacy in older patients in primary care: a northern-Italian cluster-randomised controlled trial. *BMC Geriatr.* 2021;21(1):659.
10. Mak SS, Alessi CA, Kaufmann CN, et al. Pilot RCT Testing A Mailing About Sleeping Pills and Cognitive Behavioral Therapy for Insomnia: Impact on Benzodiazepines and Z-Drugs. *Clin Gerontol.* 2024;47(3):452-463.
11. McCarthy C, Clyne B, Boland F, et al. GP-delivered medication review of polypharmacy, deprescribing, and patient priorities in older people with multimorbidity in Irish primary care (SPPiRE Study): A cluster randomised controlled trial. *PLoS Med.* 2022;19(1):e1003862.
12. Mortsiefer A, Löscher S, Pashutina Y, et al. Family Conferences to Facilitate Deprescribing in Older Outpatients With Frailty and With Polypharmacy: The COFRAIL Cluster Randomized Trial. *JAMA Netw Open.* 2023;6(3):e234723.
13. Phelan EA, Williamson BD, Balderson BH, et al. Reducing Central Nervous System-Active Medications to Prevent Falls and Injuries Among Older Adults: A Cluster Randomized Clinical Trial. *JAMA Netw Open.* 2024;7(7):e2424234.
14. Rieckert A, Reeves D, Altiner A, et al. Use of an electronic decision support tool to reduce polypharmacy in elderly people with chronic diseases: cluster randomised controlled trial. *Bmj.* 2020;369:m1822.

© 2025 Linsky AM et al. *JAMA Network Open*.

15. Rudolf H, Thiem U, Aust K, et al. Reduction of Potentially Inappropriate Medication in the Elderly. *Dtsch Arztebl Int.* 2021;118(51-52):875-882.
16. Wallis KA, Elley CR, Moyes SA, Lee A, Hikaka JF, Kerse NM. Safer Prescribing and Care for the Elderly (SPACE): a cluster randomised controlled trial in general practice. *BJGP Open.* 2022;6(1).
17. Zechmann S, Senn O, Valeri F, et al. Effect of a patient-centred deprescribing procedure in older multimorbid patients in Swiss primary care - A cluster-randomised clinical trial. *BMC Geriatr.* 2020;20(1):471.

© 2025 Linsky AM et al. *JAMA Network Open*.
