# Supplementary material for: Deprescribing in Community-Dwelling Older Adults: A Systematic Review and Meta-Analysis
Source: JAMA Netw Open. 2025 May 8;8(5):e259375. doi: 10.1001/jamanetworkopen.2025.9375 (PMC12062908; doi:10.1001/jamanetworkopen.2025.9375)
Supplement: Supplement 2. — Data Sharing Statement [file jamanetwopen-e259375-s002.pdf]

Data Sharing Statement

Linsky. Deprescribing in Community-Dwelling Older Adults. *JAMA Netw Open*. Published May 08, 2025. doi:10.1001/jamanetworkopen.2025.9375

Data
Data available: Yes
Data types: Data (not involving human participants)
How to access data: All data is reported in the evidence table
When available: With publication

Supporting Documents
Document types: None

Additional Information
Who can access the data: Data is provided in the ET
Types of analyses: N/A
Mechanisms of data availability: N/A
